# Phenotype execution and modeling architecture to support disease surveillance and real-world evidence studies: English sentinel network evaluation

**DOI:** 10.1093/jamiaopen/ooae034

**Published:** 2024-05-10

**Authors:** Gavin Jamie, William Elson, Debasish Kar, Rashmi Wimalaratna, Uy Hoang, Bernardo Meza-Torres, Anna Forbes, William Hinton, Sneha Anand, Filipa Ferreira, Rachel Byford, Jose Ordonez-Mena, Utkarsh Agrawal, Simon de Lusignan

**Affiliations:** Nuffield Department of Primary Health Care Sciences, University of Oxford, Oxford, OX2 6ED, United Kingdom; Nuffield Department of Primary Health Care Sciences, University of Oxford, Oxford, OX2 6ED, United Kingdom; Nuffield Department of Primary Health Care Sciences, University of Oxford, Oxford, OX2 6ED, United Kingdom; Nuffield Department of Primary Health Care Sciences, University of Oxford, Oxford, OX2 6ED, United Kingdom; Nuffield Department of Primary Health Care Sciences, University of Oxford, Oxford, OX2 6ED, United Kingdom; Nuffield Department of Primary Health Care Sciences, University of Oxford, Oxford, OX2 6ED, United Kingdom; Nuffield Department of Primary Health Care Sciences, University of Oxford, Oxford, OX2 6ED, United Kingdom; Nuffield Department of Primary Health Care Sciences, University of Oxford, Oxford, OX2 6ED, United Kingdom; Nuffield Department of Primary Health Care Sciences, University of Oxford, Oxford, OX2 6ED, United Kingdom; Nuffield Department of Primary Health Care Sciences, University of Oxford, Oxford, OX2 6ED, United Kingdom; Nuffield Department of Primary Health Care Sciences, University of Oxford, Oxford, OX2 6ED, United Kingdom; Nuffield Department of Primary Health Care Sciences, University of Oxford, Oxford, OX2 6ED, United Kingdom; Nuffield Department of Primary Health Care Sciences, University of Oxford, Oxford, OX2 6ED, United Kingdom; Nuffield Department of Primary Health Care Sciences, University of Oxford, Oxford, OX2 6ED, United Kingdom

**Keywords:** phenotype, systematized nomenclature of medicine, routinely collected health data, medical record systems, computerized, clinical coding

## Abstract

**Objective:**

To evaluate Phenotype Execution and Modelling Architecture (PhEMA), to express sharable phenotypes using Clinical Quality Language (CQL) and intensional Systematised Nomenclature of Medicine (SNOMED) Clinical Terms (CT) Fast Healthcare Interoperability Resources (FHIR) valuesets, for exemplar chronic disease, sociodemographic risk factor, and surveillance phenotypes.

**Method:**

We curated 3 phenotypes: Type 2 diabetes mellitus (T2DM), excessive alcohol use, and incident influenza-like illness (ILI) using CQL to define clinical and administrative logic. We defined our phenotypes with valuesets, using SNOMED’s hierarchy and expression constraint language, and CQL, combining valuesets and adding temporal elements where needed. We compared the count of cases found using PhEMA with our existing approach using convenience datasets. We assessed our new approach against published desiderata for phenotypes.

**Results:**

The T2DM phenotype could be defined as 2 intensionally defined SNOMED valuesets and a CQL script. It increased the prevalence from 7.2% to 7.3%. Excess alcohol phenotype was defined by valuesets that added qualitative clinical terms to the quantitative conceptual definitions we currently use; this change increased prevalence by 58%, from 1.2% to 1.9%. We created an ILI valueset with SNOMED concepts, adding a temporal element using CQL to differentiate new episodes. This increased the weekly incidence in our convenience sample (weeks 26-38) from 0.95 cases to 1.11 cases per 100 000 people.

**Conclusions:**

Phenotypes for surveillance and research can be described fully and comprehensibly using CQL and intensional FHIR valuesets. Our use case phenotypes identified a greater number of cases, whilst anticipated from excessive alcohol this was not for our other variable. This may have been due to our use of SNOMED CT hierarchy. Our new process fulfilled a greater number of phenotype desiderata than the one that we had used previously, mostly in the modeling domain. More work is needed to implement that sharing and warehousing domains.

## Introduction

### Moving toward shareable phenotypes

A digital phenotype refers to the rules that are applied to computerized medical records (CMRs) to identify cohorts of patients or episodes of interest.^1^ The rules are converted to computer algorithms that typically include both logical expressions and data elements such as lists of clinical codes. These algorithms are applied through computerized queries to a CMR system or other health data repository to return the desired data output.^2^

Phenotypes should be transferable between organizations and environments, to support the goals of open science.^3^ To ensure interpretability and facilitate electronic sharing of algorithms within data pipelines phenotypes should be represented in human and machine-readable formats. A number of public phenotype libraries have been developed to support the sharing and distribution of algorithms. Fourteen desiderata have been identified for the ideal phenotype library, though they are rarely even partially met.^4^

### Digital phenotyping at the Oxford-Royal College of General Practitioners-Research and Surveillance Centre

The RSC collects CMR data from over 1800 English primary care practices for surveillance, research, quality improvement, and educational purposes.^5^ These pseudonymized data are securely stored in a trusted research environment (TRE).^5^ The RSC has issued weekly disease surveillance reports to the UK Health Security Agency (UKHSA) and its predecessors for over 50 years, and conducts chronic disease CMR research. Development of digital phenotypes, or phenotyping, is pivotal for the work undertaken by the RSC.^6^

**Figure 1.**
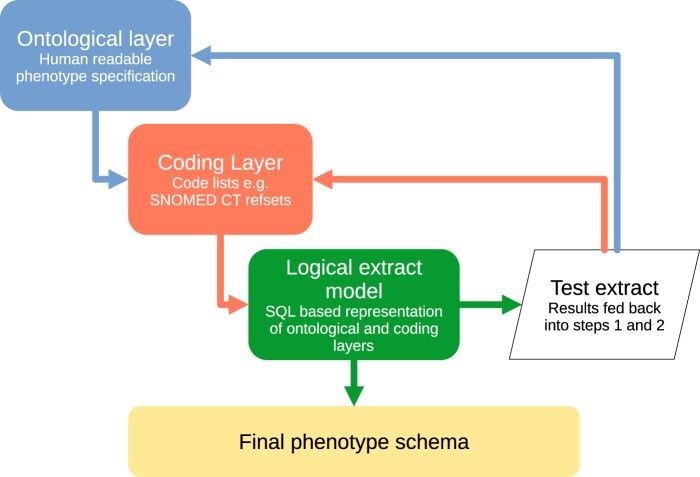
Existing 3-layered approach for defining phenotypes at the RSC. Ontological layer: human-readable phenotype specification, may utilize diagnostic criteria, symptom examination findings, test result, and therapies. Coding layer: Creation of code lists typically using SNOMED CT. Logical extract model: structured query language (SQL) scripts are manually developed based on ontological and coding layer. Test extract: Assessed for veracity modifications are fed back to ontological and coding layers. The phenotype schema can then be finalized.

At the RSC, we use a 3-layered approach to phenotyping shown in Figure 1.^7^ Ontological layer: a human-readable description of the key concepts and relationships of a phenotype. Coding layer: where the conceptual reasoning is used to generate lists of clinical codes. Our code lists are typically based on Systematised Nomenclature of Medicine (SNOMED) Clinical Terms (CT) as this is the mandated terminology used in United Kingdom primary care.^8^ The United Kingdom edition of SNOMED CT is published twice a year by NHS England.^9^

We will try to identify any existing relevant lists of codes at the Primary Care Domain Reference Set Portal.^10^ SNOMED CT code lists are typically formatted as refsets. We develop refsets using our in house software tool, the “SNOMED helper tool,” shown in Figure S1. Logical data extract layer: where Structured Query Language (SQL) queries are manually written using logical expressions combined with the refsets to extract the desired data. The data is subsequently tested for face validity and compared with other data sources.

We have previously defined important phenotypes such as pregnancy, chronic kidney disease, social prescribing interventions, and long COVID with Web Ontology Language (OWL) using the Protégé software.^11–13^ These phenotypes were then uploaded to the public phenotype libraries Bioportal and PhenoFlow.^14^

### Case for changing our approach to developing phenotypes

We are changing our approach to developing phenotypes as, firstly, we found OWL excellent for semantic precision, but sometimes challenging to align with clinical and project reasoning. Secondly, in the coding layer we had previously defined refsets “extensionally.” This is where each code is explicitly listed as a member of the sets. We now plan to make better use of the SNOMED expression constraint language (ECL).^15–17^ SNOMED CT is designed to be machine processable and its ECL allows a rule-based or “intensional” approach to developing refsets that utilize SNOMED’s polyhierarchical subtypes and supertypes.^14^ The logic used to define intensional refsets is more explicit.

### Evaluation of Phenotype Execution and Modelling Architecture

Phenotype Execution and Modelling Architecture is a standards-based, modular architecture for developing phenotype algorithms, including their validation, execution, and dissemination developed for real-world studies using CMR data.^18^ PhEMA uses aspects of Health Level 7’s (HL7) Fast Healthcare Interoperability Resources (FHIR) framework including the use of Clinical Quality Language (CQL). CQL is a structured way of expressing phenotype logic and code lists and allows expression and sharing of phenotype logic, it is designed to be human and machine readable.^19^^,^^20^

This study aimed to evaluate PhEMA’s CQL-based logic model and creation of HL7 FHIR valuesets, into the RSC’s 3-layered approach to phenotyping, replacing OWL or unstructured phenotype definitions. We adapted our approach to incorporate PhEMA elements and then aimed to develop phenotypes for 3 purposively selected use cases: type 2 diabetes mellitus (T2DM), incident influenza-like illness (ILI), and excessive alcohol consumption.

## Method

### The three phenotype use cases

We selected 3 dissimilar use cases that we anticipated would require different logic models. The 3 we selected were: (1) T2DM, (2) Excessive alcohol use, and (3) Incident cases of ILI. We compared the new phenotypes with those produced using our current process, and where a variable is new looked for external validation.

#### Use case 1: type 2 diabetes mellitus

We chose the T2DM phenotype due to our extensive experience with CMR-based T2DM studies and its frequent use as a covariate in other research.^21–23^ Our involvement extends to examining misdiagnosis, miscoding, and misclassification within CMRs.^24–26^ Phenotyping T2DM is challenging because the diagnosis can be derived through numerous clinically coded events, including diagnostic codes, laboratory results, or the prescribing of medication. Additionally, T2DM may resolve through lifestyle interventions and bariatric surgery, but there is potential later relapse.^27–29^ The RSC has a well-established but complex phenotyping algorithm based on our existing 3-layered approach.^30^ To evaluate PhEMA’s potential integration, however, we will use a simplified version of our current T2DM phenotype.

#### Use case 2: excessive alcohol use

The alcohol use case phenotype is an exemplar of a sociodemographic risk factor. We identified a variety of definitions of excessive alcohol use and chose one based on consumption levels rather than questionnaires about the effects of drinking. The National Health Service (NHS) defines excessive consumption of alcohol as more than 14 units of alcohol per week.^31^

#### Use case 3: incident influenza-like illness

The World Health Organization (WHO) recommends surveillance of the respiratory clinical syndrome ILI as a marker of community spread of respiratory infections. Tracking the incidence of ILI is important because in respiratory surveillance systems around half of ILI clinical cases have laboratory confirmed influenza.^32^ Alongside other surveillance indicators ILI surveillance should be part of the mosaic of measures used to flag an emergent pandemic.^33^^,^^34^ The RSC provides weekly ILI incidence figures to UKHSA in its weekly report.

### Modifying our 3-step ontological process for defining a phenotype in real-world data

**Figure 2.**
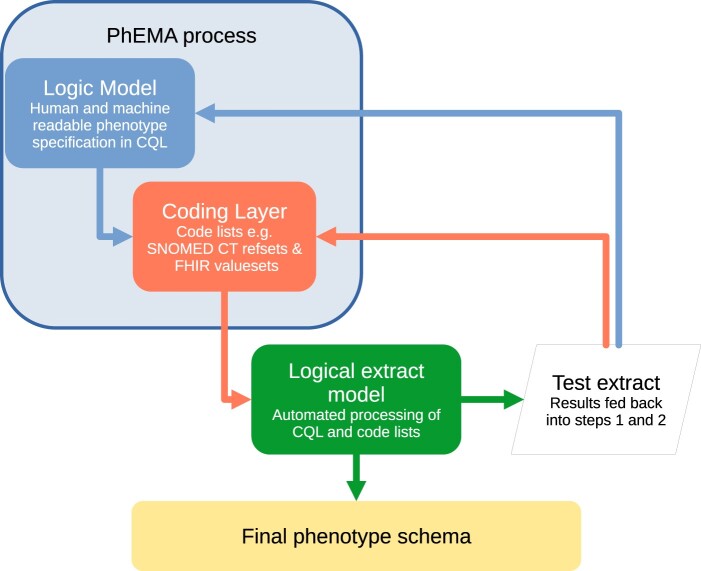
Revised 3-step model for developing phenotypes at the RSC. Logic model: Define clinical and operational logic using clinical quality language (CQL). Coding layer: Create intensional refsets using in-house SNOMED CT helper tool (Figure S1). SNOMED refsets are converted to fast healthcare interoperability resources (FHIR) valuesets for CQL compatibility. Logical extraction model: Automated processing of machine-readable PhEMA output to extract data from CMR. Test extract: Assessed for veracity modifications are fed back to PhEMA layers. Internal and external validation processes.

Our modified 3-step approach to embrace PhEMA principles is shown in Figure 2. The logic model and coding layer use the PhEMA processes with logic model expressed in CQL. The PhEMA logic model includes operational factors in addition to clinical concepts. Operational factors describe non-clinical factors that influence CMR coding and may relate to the data entry environment or systems of practice. For instance, variations in the use of specific codes might be influenced by contractual requirements and incentives; this context is essential for our approach. Additionally, we alter the criteria to adjust the sensitivity or specificity of the extraction according to the aims of the study.^35^ The logical data extraction step is external to the PhEMA process. Here we generate the data output and check the internal and external validity of new phenotypes.

### Step 1: describing the logic model using clinical quality language

We used the PhEMA work bench to define the phenotype logic model for each of our 3 use cases in CQL.^36^ Subsequently we used Visual Studio Code from Microsoft, with the CQL extension which provides syntax highlighting and debugging tools.^37^^,^^38^ Our CQL code is based resources developed in the FHIR and PhEMA helper libraries.^39^^,^^40^ The resources produced under the PhEMA and Clinical Quality Framework (CQF), including CQL, contain a set of examples that can be used to generate the majority of commonly used phenotypes. We found the HL7 Confluence community workspace, the most accessible route to get started.^41^

### Step 2: defining the FHIR valuesets and SNOMED CT refsets

SNOMED refsets were initially created using our SNOMED Helper Tool (Figure S1). SNOMED’s ECL allows sets of concepts to be defined intensionally from their relationships.^42^ Most often this is a hierarchical relationship where a high-level concept (known as a supertype) is explicitly defined and all lower level concepts (known as subtypes), whose meanings are subsumed by the higher concept, are included by inference.^13–15^ Conversely, concepts may be excluded by specifying a minus supertype and none of its subtypes will be included. The SNOMED ECL functions we used included “Child or Self of” (written childOrSelfOf) where a clinical term and its child codes are included.^43^ SNOMED refsets were then converted to FHIR valuesets.

FHIR valuesets are a set of clinical terms and associated metadata that are compatible with multiple code systems, including intensional SNOMED CT expressions or refsets. Metadata can include the coding or classification system used, the valueset provenance and lifecycle management, such as marking a valueset as experimental.^44^ A FHIR valueset can be marked as a draft, or at end of life, be formally retired whilst preserving the content.^45^

### Step 3: describing the logical extract model

We used the developed phenotypes to extract the associated cohort of patients from the RSC SQL-based CMR. We did this by manually converting CQL phenotype logic to SQL. The RSC CMR is a custom data store containing data extracted from general practice Electronic Health Record (EHR) systems in England and does not currently present a common data model such as OMOP. The process of translation and validation is therefore manual. We plan to automate this step in the future to take full advantage of the machine-readable nature of CQL. From the extracted data, we then calculated summary statistics for each of the use cases.

#### Type 2 diabetes mellitus

We compared the prevalence of T2DM in the current adult (>18 years) RSC population to that of a recent cohort that used our previous phenotype. Additionally, we compared the prevalence in all age groups to that of the estimated UK diabetes prevalence using data from Diabetes UK and the Office for National Statistics (ONS).

#### Excessive alcohol use

We assessed excessive alcohol consumption prevalence using qualitative SNOMED codes, then combined them with quantitative SNOMED codes, specifically the units of alcohol consumed, and determined relative percentage increase in prevalence with the addition of quantitative codes.

#### Influenza-like illness

We compared the incidence of ILI for ISO weeks 26-38 of 2023, the most recent data available, using both the old and new phenotypes. We used these dates as a convenience sample. We report the median, lower, and upper quartiles of the weekly incidence of ILI by age band for this period. The age bands used are consistent with those presented in the weekly surveillance report to UKHSA.

### Evaluation against desiderata

We will use the described desiderata^4^ to evaluate the progress toward an ideal phenotype library. There are 7 which are relevant to our revised process. Four of these desiderata concern phenotype authoring:

- Support modeling languages.
- Support natural language processing-based and machine learning-based definitions.
- Support multi-dimensional descriptions.
- Support modular relationships.

A further 3 desiderata concern validation:

- Support a defined validation process.
- Automate multiple validation techniques.
- Enable feedback.

### Ethical considerations

The variables curated in this work are all required for our sentinel surveillance, approved under Regulation 3 of the Health Service (Control of Patient Information) Regulations 2002, and reviewed and approved annually by the UKHSA Caldicott Guardian.^46^

## Results

### Use case 1: type 2 diabetes mellitus

#### Logic model

**Figure 3.**
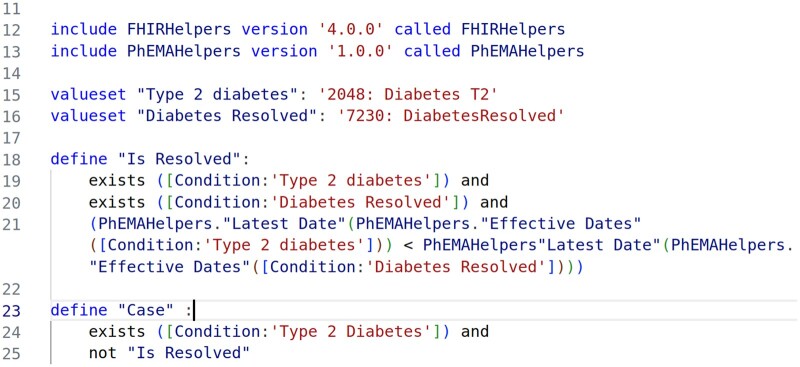
Clinical quality language (CQL) logic model for our type 2 diabetes mellitus (T2DM) phenotype. Individual is coded as resolved if resolved date is greater than diagnosis date.

Clinical terms that inferred a diagnosis of T2DM were identified intensionally from the SNOMED hierarchy. For this simplified diabetes phenotype, we did not use blood glucose levels or prescription of medication to indicate a diagnosis of T2DM. We also identified individuals whose diabetes had resolved and excluded them from the final cohort. For operational logic we included process of care codes which imply a diagnosis of T2DM, for example, “Type 2 DM review,” (STCID: 279321000000104). The CQL code can be seen in Figure 3.

#### Coding layer

We created 2 SNOMED CT refsets, the first consisting of codes implying the diagnosis of diabetes and the second implying that the diagnosis had resolved. The first refset included the diagnostic codes such as “Diabetes mellitus type 2” (SCTID: 44054006) and all of its descendant codes. We additionally used single relevant clinical terms such as “Type 2 diabetic on insulin” (SCTID: 24471000000103) and other concepts and their relevant child concepts. Subtypes make up the bulk of the definition—97% of the concepts in the final, expanded, extensional set arose from the “childOrSelfOf” definitions (Textbox S1).

Operationally, diabetes is also part of the Quality and Outcomes Framework so practices have a financial incentive to use specific codes which are published by the NHS.^10^ We ensured that all these concepts were included in the valueset. The contractual requirements for coding have led to a standard of coding by practices which has allowed a simpler phenotype.

#### Logical extract model

In individuals over 18 years of age, the RSC CMR’s diabetes prevalence using the new phenotype was 7.3%. This is slightly higher than the 7.2% prevalence from a 2021 cohort study on sodium-glucose co-transporter-2 inhibitor prescriptions in primary care, which utilized the old RSC phenotype and 2019 RSC data.^47^ For a national comparison, we incorporated data for those under 18. The prevalence across all age groups with the new phenotype was 5.9%, in contrast to an approximate 5.3% derived from Diabetes UK and the ONS data.^48^

Although our database only contains coded data we were able to run the phenotype in the EHR in the practice where G.J. is a general practitioner. One hundred patients identified by the phenotype were selected randomly for a manual review of the record. Two patients were found to have identified in error due to miscoding of the diabetes type in the record. One further patient had resolution of diabetes which had not been coded and one patient had remission coded with a concept which was not included in the valueset. Overall, this gives a positive predictive value (PPV) of 0.96 for this sample in a single practice.

### Use case 2: excessive alcohol use

#### Logic model

**Figure 4.**
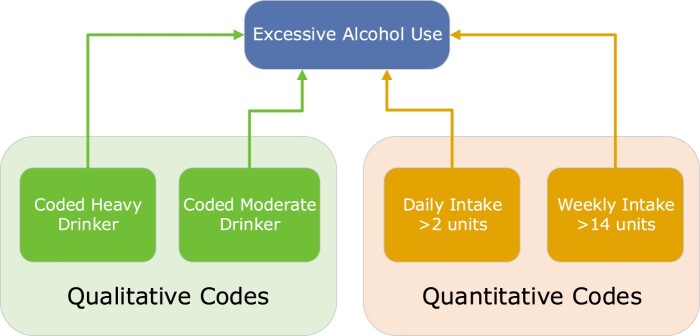
Clinical concepts used to define the excessive alcohol use valueset. Including qualitative codes and quantitative codes that represent the number of units of alcohol consumed in the specified time period.

Using only clinical logic we identified individuals with evidence of consumption of greater than 14 units of alcohol per week. Firstly, we looked for qualitative codes with a rubric that includes the specific number of units consumed, for example, “moderate drinker 3-6u/day,” (SCTID: 160576006). We then identified codes allowing input of qualifying values, in this case the specific number of units consumed by an individual. We demonstrate our final logical model for excessive alcohol use (Figure 4), with the CQL logic in a supplementary file.

#### Coding layer

We created 4 valuesets which combined to produce our phenotype, 2 containing qualitative codes and 2 contained codes with associated qualifying values (quantitative codes). The qualitative value sets contain concepts from the “findings” section of the SNOMED CT hierarchy. With 1 valueset defining heavy drinkers including “Heavy drinker 7-9u/day” and “Very heavy drinker >9u/day” codes. The second valueset contains the single concept for moderate drinker. Two valuesets will contain “observable entity” concepts from the UK SNOMED CT Extension: “Alcohol units consumed per day” and “Alcohol units consumed per week.” The CQL for excessive alcohol consumption can be seen in Textbox S2.

#### Logical extract model

The overall prevalence of excessive alcohol consumption in the RSC CMR using only qualitative SNOMED codes was 1.2%. The addition of quantitative codes to the phenotype increased the prevalence to 1.9%, a 58.3% relative increase although this is still considerably below the Health Survey data for England which reported 21% of adults drinking more than 14 units weekly.^49^Table 1 shows the breakdown of prevalence by age band. The increase in prevalence at age 40 is likely due to the NHS Health Check which includes questions about alcohol use and is offered to all registered patients every 5 years from age 40 to age 75.^50^ It seems that excessive alcohol use is being recorded in EHRs without a diagnostic code of excess entered, as might be expected if a data entry template is used. These may represent patients who are less likely to receive interventions aimed at modifying their alcohol use.

**Table 1.** Prevalence of excessive alcohol consumption in the RSC in those 16 years or older.

Age band	Qualitative codes^a^	Combined codes^b^	% change^c^
All (≤16 years)	1.45%	2.30%	+58.31%
16-40 years	0.54%	0.31%	+77.34%
41-65 years	3.05%	1.86%	+64.54%
>65 years	4.37%	2.95%	+48.26%

a Prevalence using only qualitative codes.

b Prevalence using qualitative and quantitative codes.

c The percentage increase in prevalence through use of combined codes.

### Use case 3: influenza-like illness

#### Logic model

As ILI is an acute clinical syndrome that may recur multiple times in the same individual, we aimed to identify weekly incident cases. This contrasts with the previous use cases in which we were identifying the prevalence of a condition. We developed an approach to distinguish between new and repeat presentations of the same ILI episode using the PhEMA framework. We identified ILI clinical terms recorded more than 28 days, from the previous recorded event (Supplementary file ILI.cql). As this requires use of date values within CQL we used the PhEMAHelpers library which contains functions to simplify representation of dates.

#### Coding layer

We created a single valueset from the SNOMED CT identifying conceptually different areas within SNOMED CT which met our clinical criteria for ILI. These included the following codes and their subtypes: “ILI,” “Influenza-like symptoms,” and “Influenza.” Additionally, we excluded high-level concepts within the hierarchy such as “Healthcare associated infections” which excludes “Healthcare associated influenza disease” from within the “influenza” parent code.

#### Logical extract model

Between ISO weeks 26 and 38 of 2023, the median weekly incidence of ILI using the new phenotype definition was 1.11 cases per 100 000 people. In contrast, using the old phenotype definition, it was 0.95 cases per 100 000 people. Table 2 shows the weekly incidence of ILI by age band. The most notable change to the phenotype was the increased number of concepts described by the intensional nature of the valueset which has increased the number of cases identified.

**Table 2.** Median weekly incidence of ILI in the RSC CMR for ISO weeks 26-38 of 2023 using both the new and old phenotypes.

Age band	New phenotype	Old phenotype
All ages	1.11 (0.66-1.44)	0.95 (0.60-1.25)
<15 years	0.54 (0.34-0.68)	0.53 (0.25-0.69)
15-64 years	1.51 (1.31-1.65)	1.41 (1.16-1.54)
>65 years	1.16 (1.00-1.22)	1.02 (0.68-1.16)

Values denote medians, with lower and upper quartiles enclosed in brackets.

### Assessment against phenotype desiderata

We assessed the methods against the desiderata for authoring and validation (Table 3). The new methodology met 3 out of 4 of the authoring criteria. We had not implemented Natural Language Processing (NLP) or Machine Learning (ML). There was some movement toward the validation criteria although none were fully met.

**Table 3.** Evaluation of desiderata against old and new processes.

Desiderata	Previous method	New method
**Authoring**		
Modeling languages	SNOMED CT ECL for valuesets only^a^	Yes, using CQL for logic and ECL for valuesets^b^
Multi-dimensional descriptions	Informally and separate from implementation	More formally within CQL
NLP and ML definitions	No	No
Modular relationships	No	CQL files may be used as libraries
**Validation**		
Defined validation process	Yes	Yes—multiple methods used. No access to full records for patient record review
Automate validation techniques	No	No, but increased potential to do so
Enable feedback	Yes, internally	Yes, internally

a Expression Constraint Language

b Clinical Quality Language

## Discussion

### Principal findings

We have demonstrated that we can effectively use PhEMA to curate phenotypes—creating valuesets and more shareable logic. SNOMED refsets created intensionally take advantage of SNOMED’s consistent hierarchy and ECL. Using CQL to express clinical and operational logic was comprehensible to clinicians and epidemiologists in our team and will be adopted. We have demonstrated this across 3 different use cases. Across all these use cases the case acquisition increased. We anticipated that the prevalence of excessive alcohol would increase, as we were adding qualitative clinical terms. However, it was not anticipated for T2DM where we added the capability to go into remission or in ILI where we added a temporal constraint to help ensure that we only counted incident cases. Our interpretation is that the intension use of SNOMED’s ECL accounted for this increase.

Our new approach achieves 3 out of 4 of the authoring desiderata and takes us closer to the achievement of validation desiderata.

### Implications of the finding

We will migrate our approach to curating variables from OWL, where we were more challenged to express clinical logic, to PhEMA, and CQL. The separation of clinical logic from administrative, largely health system constraints, was considered helpful. Standardizing our valuesets using the FHIR metadata improves our ability to share and version control our curated variables. This is a step toward phenotypes that are readily machine processable. Intension use of SNOMED’s ECL may be important in ensuring that all relevant clinical terms within that hierarchy are captured.

### Comparison with the literature

There have been several attempts to allow sharing of phenotypes.^51–53^ These have used their own formats for expressing the criteria for extracting data from the medical record. Logic is typically described in free text or expressed using general scripting languages with an implementation which is closely tied to the underlying data structure and storage used by the researcher.

In the United Kingdom the leading organizations promoting shared phenotypes and code lists have been Health Data Research UK and OpenSafely, respectively.^54^^,^^55^ However, neither of these include interoperable expressions of clinical logic.

The US National Library of Medicine distributes valuesets through the Valueset Authority Center,^56^ but the NHS England Terminology Server currently lacks the former’s scope.^57^ Health records and SNOMED CT differ significantly between in the United States and the NHS.

We unambiguously accepted the rigor of the SNOMED hierarchy, and whilst vastly better than the inconsistencies of the United Kingdom’s previous Read codes, there are limitations and questions as to whether they are sufficiently quality assured.^58^^,^^59^

### Strengths and limitations

The research group have had long experience of curating variables across the range of clinical terminologies used in the English NHS. We have shown that phenotypes can be expressed and distributed in a way that is comprehensible to both humans and computers and that implementing the PhEMA Framework is technically and practically feasible.

Whilst we have met 3 of the desiderata that Chapman^4^ expressed for the creation of a next generation phenotype library, the many gaps that remain are a limitation of this process. We have only started to address the sharing and warehousing desiderata and this will become the focus of our ongoing work. We demonstrated this process using the hierarchies of SNOMED CT, but it would apply to less ontologically complex terminologies such as ICD or OPCS. Our conclusions about the interpretability of CQL are based on the opinions of the authors and no formal consensus building or qualitative assessment was conducted.

We will need considerable effort to convert our large library of curated variables into PhEMA format. An open valueset authority in the United Kingdom, as already exists in United States, would enhance our ability to collaborate and share valuesets.

## Conclusion

We have shown that we can express phenotypes using the principles of the PhEMA framework for the identification of patients with chronic disease, the assessment of observations and for surveillance of communicable disease using international health data standards.

It was feasible and practical to distribute FHIR valuesets using intensional definitions which can be compiled to extensional lists by terminology servers. This approach also increased case finding.

We have described the logic unambiguously using CQL, a FHIR specification, in a way which is understandable for humans as well as potentially computable.

## Supplementary Material

ooae034_Supplementary_Data

## Data Availability

The CQL scripts in this study can be downloaded from https://github.com/gjamie/Phenotypes. The valuesets are available from the NHS Terminology Server https://ontology.nhs.uk.

## References

[1] Brandt PS , Kho A, Luo Y, et al Characterizing variability of electronic health record-driven phenotype definitions. J Am Med Inform Assoc. 2023;30(3):427-437. 10.1093/jamia/ocac235 36474423 PMC9933077

[2] Richesson RL , Hammond WE, Nahm M, et al Electronic health records based phenotyping in next-generation clinical trials: a perspective from the NIH health care systems collaboratory. J Am Med Inform Assoc. 2013;20(e2):e226-e231. 10.1136/amiajnl-2013-001926 23956018 PMC3861929

[3] UNESCO. 2022. Understanding open science. Accessed October 2023. https://unesdoc.unesco.org/ark:/48223/pf0000383323

[4] Chapman M , Mumtaz S, Rasmussen LV, et al Desiderata for the development of next-generation electronic health record phenotype libraries. Gigascience. 2021;10(9):giab059. 10.1093/gigascience/giab059 34508578 PMC8434766

[5] de Lusignan S , Jones N, Dorward J, et al The Oxford royal college of general practitioners clinical informatics digital hub: protocol to develop extended COVID-19 surveillance and trial platforms. JMIR Public Health Surveill. 2020;6(3):e19773. 10.2196/19773 32484782 PMC7333793

[6] Gu X , Watson C, Agrawal U, et al Postpandemic sentinel surveillance of respiratory diseases in the context of the world health organization mosaic framework: Protocol for a development and evaluation study involving the english primary care network 2023-2024. JMIR Public Health Surveill. 2024;10:e52047. 10.2196/52047 38569175 PMC11024753

[7] de Lusignan S. In this issue: ontologies a key concept in informatics and key for open definitions of cases, exposures, and outcome measures. J Innov Health Inform. 2015;22(2):170. 10.14236/jhi.v22i2.170 26245238

[8] NHS Digital SNOMED CT. Accessed October 29, 2023. https://digital.nhs.uk/snomed-ct

[9] NHS England. Technology reference update distribution. Accessed January 2, 2024. https://isd.digital.nhs.uk/trud/users/guest/filters/0/home.

[10] NHS England Primary Domain Reference Set Portal. Accessed January 2, 2024. https://digital.nhs.uk/data-and-information/data-collections-and-data-sets/data-collections/quality-and-outcomes-framework-qof/quality-and-outcome-framework-qof-business-rules/primary-care-domain-reference-set-portal

[11] Liyanage H , Williams J, Byford R, et al Ontology to identify pregnant women in electronic health records: primary care sentinel network database study. BMJ Health Care Inform. 2019;26(1):e100013. 10.1136/bmjhci-2019-100013 PMC7062332 31272998

[12] Cole NI , Liyanage H, Suckling RJ, et al An ontological approach to identifying cases of chronic kidney disease from routine primary care data: a cross-sectional study. BMC Nephrol. 2018;19(1):85. 10.1186/s12882-018-0882-9 29636024 PMC5894169

[13] Jani A , Liyanage H, Okusi C, et al Using an ontology to facilitate more accurate coding of social prescriptions addressing social determinants of health: feasibility study. J Med Internet Res. 2020;22(12):e23721. 10.2196/23721 33306032 PMC7762682

[14] Mayor N , Meza-Torres B, Okusi C, et al Developing a long COVID phenotype for postacute COVID-19 in a national primary care sentinel cohort: observational retrospective database analysis. JMIR Public Health Surveill. 2022;8(8):e36989. 10.2196/36989 35861678 PMC9374163

[15] Benson T , Grieve G. SNOMED CT concept model. In: Principles of Health Interoperability. Health Information Technology Standards. Springer; 2016:173-187. ISBN 9783319303680. 10.1007/978-3-319-30370-3_10

[16] Willett DL , Kannan V, Chu L, et al SNOMED CT concept hierarchies for sharing definitions of clinical conditions using electronic health record data. Appl Clin Inform. 2018;9(3):667-682. 10.1055/s-0038-1668090 30157499 PMC6115233

[17] Giménez-Solano VM , Maldonado JA, Boscá D, et al Definition and validation of SNOMED CT subsets using the expression constraint language. J Biomed Inform. 2021;117:103747. 10.1016/j.jbi.2021.103747 33753269

[18] Rasmussen LV , Kiefer RC, Mo H, et al A modular architecture for electronic health record-driven phenotyping. AMIA Jt Summits Transl Sci Proc. 2015;eCollection 2015:147-151. 26306258 PMC4525215

[19] Brandt PS , Kiefer RC, Pacheco JA, et al Toward cross-platform electronic health record-driven phenotyping using clinical quality language. Learn Health Syst. 2020;4(4):e10233. 10.1002/lrh2.10233 33083538 PMC7556419

[20] Pacheco JA , Rasmussen LV, Kiefer RC, et al A case study evaluating the portability of an executable computable phenotype algorithm across multiple institutions and electronic health record environments. J Am Med Inform Assoc. 2018;25(11):1540-1546. 10.1093/jamia/ocy101 30124903 PMC6213083

[21] Feher MD , Munro N, Russell-Jones D, et al Novel diabetes subgroups. Lancet Diabetes Endocrinol. 2018;6(6):439. 10.1016/S2213-8587(18)30126-8 29803263

[22] Whyte MB , Joy M, Hinton W, et al Early and ongoing stable glycaemic control is associated with a reduction in major adverse cardiovascular events in people with type 2 diabetes: a primary care cohort study. Diabetes Obes Metab. 2022;24(7):1310-1318. 10.1111/dom.14705 35373891 PMC9320871

[23] de Lusignan S , McGovern A, Hinton W, et al Barriers and facilitators to the initiation of injectable therapies for type 2 diabetes mellitus: a mixed methods study. Diabetes Ther. 2022;13(10):1789-1809. 10.1007/s13300-022-01306-z 36050586 PMC9500132

[24] Stone MA , Camosso-Stefinovic J, Wilkinson J, et al Incorrect and incomplete coding and classification of diabetes: a systematic review. Diabet Med. 2010;27(5):491-497. Erratum in: *Diabet Med*. 2010;27(6):732. 10.1111/j.1464-5491.2009.02920.x 20536944

[25] de Lusignan S , Khunti K, Belsey J, et al A method of identifying and correcting miscoding, misclassification and misdiagnosis in diabetes: a pilot and validation study of routinely collected data. Diabet Med. 2010;27(2):203-209. 10.1111/j.1464-5491.2009.02917.x 20546265

[26] de Lusignan S , Sadek N, Mulnier H, et al Miscoding, misclassification and misdiagnosis of diabetes in primary care. Diabet Med. 2012;29(2):181-189. 10.1111/j.1464-5491.2011.03419.x 21883428

[27] Ried-Larsen M , Johansen MY, MacDonald CS, et al Type 2 diabetes remission 1 year after an intensive lifestyle intervention: a secondary analysis of a randomized clinical trial. Diabetes Obes Metab. 2019;21(10):2257-2266. 10.1111/dom.13802 31168922 PMC6772176

[28] de Oliveira VLP , Martins GP, Mottin CC, et al Predictors of long-term remission and relapse of type 2 diabetes mellitus following gastric bypass in severely obese patients. Obes Surg. 2018;28(1):195-203. PMID:28770424. 10.1007/s11695-017-2830-3 28770424

[29] Arterburn DE , Bogart A, Sherwood NE, et al A multisite study of long-term remission and relapse of type 2 diabetes mellitus following gastric bypass. Obes Surg. 2013;23(1):93-102. 10.1007/s11695-012-0802-1 23161525 PMC4641311

[30] McGovern A , Hinton W, Correa A, et al Real-world evidence studies into treatment adherence, thresholds for intervention and disparities in treatment in people with type 2 diabetes in the UK. BMJ Open. 2016;6(11):e012801. 10.1136/bmjopen-2016-012801 PMC5168506 27884846

[31] National Health Service (NHS). Overview: alcohol misuse. Accessed July 2023. https://www.nhs.uk/conditions/alcohol-misuse

[32] Hammond A , Kim JJ, Sadler H, et al Influenza surveillance systems using traditional and alternative sources of data: a scoping review. Influenza Other Respir Viruses. 2022;16(6):965-974. 10.1111/irv.13037 36073312 PMC9530542

[33] World Health Organization. 2013. Global epidemiological surveillance standards for influenza. Accessed August 2023. https://apps.who.int/iris/rest/bitstreams/1210991/

[34] Mott JA , Bergeri I, Lewis HC, et al Facing the future of respiratory virus surveillance: the mosaic surveillance framework. Influenza Other Respir Viruses. 2023;17(3):e13122. 10.1111/irv.13122 36970570 PMC10030356

[35] de Lusignan S , Sun B, Pearce C, et al Coding errors in an analysis of the impact of pay-for-performance on the care for long-term cardiovascular disease: a case study. Inform Prim Care. 2014;21(2):92-101. 10.14236/jhi.v21i2.62 24841410

[36] GitHub. Phema-workbench-app. Accessed August 2023. https://github.com/PheMA/phema-workbench-app

[37] Microsoft Visual Studio Code. Accessed August 2023. https://code.visualstudio.com

[38] Visual Studio Marketplace. Clinical quality language (CQL) clinical quality framework. Accessed August 2023. https://marketplace.visualstudio.com/items?itemName=cqframework.cql

[39] GitHub. Community projects. Clinical quality framework, clinical quality language. Accessed August 2023. https://github.com/cqframework/clinical_quality_language/wiki/Community-Projects

[40] HL7 FHIR Foundation Enabling health interoperability through FHIR. Clinical Quality Framework Common FHIR Assets. Accessed August 2023. http://fhir.org/guides/cqf/common/history.html

[41] Confluence (HL7 Community workspace). Clinical quality framework. Accessed August 2023. https://confluence.hl7.org/display/CQIWC/Clinical+Quality+Framework

[42] International Health Terminology Standards Development Organisation. Expression constraint language – specification and guide. Accessed July 25, 2023. https://confluence.ihtsdotools.org/display/DOCECL

[43] SNOMED CT. MCRM maintenance tool user guide. Expression constraint language (ECL). Accessed August 2023. https://confluence.ihtsdotools.org/pages/viewpage.action?pageId=110341487

[44] ValueSet FHIR 5.0.0. Accessed August 2023. https://www.hl7.org/fhir.valueset.html

[45] Health Level 7 (HL7). Retired value sets. Accessed August 2023. https://build.fhir.org/ig/HL7/UTG/valuesets-retired.html

[46] Taylor MJ. Legal bases for disclosing confidential patient information for public health: distinguishing between health protection and health improvement. Med Law Rev. 2015;23(3):348-374. 10.1093/medlaw/fwv018 25995294 PMC4533707

[47] Hinton W , Feher MD, Munro N, et al Prescribing sodium-glucose co-transporter-2 inhibitors for type 2 diabetes in primary care: influence of renal function and heart failure diagnosis. Cardiovasc Diabetol. 2021;20(1):130. 10.1186/s12933-021-01316-4 34183018 PMC8237469

[48] Diabetes UK. 2023. How many people in the UK have diabetes? Accessed October 2023. https://www.diabetes.org.uk/professionals/position-statements-reports/statistics

[49] NHS England. 2021. Health survey for England. Accessed November 7, 2023. https://digital.nhs.uk/data-and-information/publications/statistical/health-survey-for-england

[50] Robson J , Dostal I, Sheikh A, et al The NHS health check in England: an evaluation of the first 4 years. BMJ Open. 2016;6(1):e008840. 10.1136/bmjopen-2015-008840 PMC4735215 26762161

[51] Kirby JC , Speltz P, Rasmussen LV, et al PheKB: a catalog and workflow for creating electronic phenotype algorithms for transportability. J Am Med Inform Assoc. 2016;23(6):1046-1052. 27026615 10.1093/jamia/ocv202 PMC5070514

[52] HDRUK Phenotype Library. Accessed July 25, 2023. https://phenotypes.healthdatagateway.org/

[53] Chapman M , Rasmussen LV, Pacheco JA, Curcin V. Phenoflow: A microservice architecture for portable workflow-based phenotype definitions. AMIA Jt Summits Transl Sci Proc. 2021;2021:142-151. 34457128 PMC8378606

[54] Health Data Research UK (HDRUK). Phenotype library. Accessed August 2023. https://phenotypes.healthdatagateway.org/

[55] OpenSafey. OpenCodelists. Accessed October 2023. https://www.opencodelists.org/

[56] National Library of Medicine. Value set authority centre. Accessed July 25, 2023. https://vsac.nlm.nih.gov/

[57] NHS England. The NHS England terminology server. Accessed January 2024. https://digital.nhs.uk/services/terminology-server

[58] Rodrigues JM , Schulz S, Mizen B, Trombert B, Rector A. Scrutinizing SNOMED CT's ability to reconcile clinical language ambiguities with an ontology representation. Stud Health Technol Inform. 2018;247:910-914. 29678093

[59] Rodrigues JM , Schulz S, Mizen B, Rector A, Serir S. Is the application of SNOMED CT concept model sufficiently quality assured?. AMIA Annu Symp Proc. 2017;2017:1488-1497. 29854218 PMC5977666

