# Supplementary material for: Phenotype execution and modeling architecture to support disease surveillance and real-world evidence studies: English sentinel network evaluation
Source: JAMIA Open. 2024 May 10;7(2):ooae034. doi: 10.1093/jamiaopen/ooae034 (PMC11087727; doi:10.1093/jamiaopen/ooae034)
Supplement: ooae034_Supplementary_Data [file ooae034_supplementary_data.zip › heavydrinker.cql.docx]

/**

* Orchid Phenotype

*

* Name : Influenza Like Illness

* ID : 1

* Url : https://orchid.phc.ox.ac.uk/phenotype/138

*/

library "Influenza Like Illness" version '1.0.0'

using FHIR version '4.0.0'

include FHIRHelpers version '4.0.0' called FHIRHelpers

include PhEMAHelpers version '1.0.0' called PhEMAHelpers

valueset "Influenza Like Illness": '1020: Influenza-Likeillness-WRpt'

context Patient

define function "Previous Episodes in 14 days"(

dt System.DateTime

):

[Condition: "Influenza Like Illness"] C

where

dt before PhEMAHelpers."Effective Date"(C)

and

difference in days between dt

and

PhEMAHelpers."Effective Date"(C) < 14

return

PhEMAHelpers."Effective Date"(C)

define function "New ILI Episodes":

[Condition: "Influenza Like Illness"] ILI1

where

Count(

"Previous Episodes in 14 day"(PhEMAHelpers."Effective Date"(ILI1))

) =0

define "case":

"New ILI Episodes"
