# Supplementary material for: Phenotype execution and modeling architecture to support disease surveillance and real-world evidence studies: English sentinel network evaluation
Source: JAMIA Open. 2024 May 10;7(2):ooae034. doi: 10.1093/jamiaopen/ooae034 (PMC11087727; doi:10.1093/jamiaopen/ooae034)

## **Supplementary file**

**Figure S1:** Helper Tool used to facilitate the development of SNOMED CT refsets

The upper figure displays the type two diabetes (T2DM) refset. The lower annotated figure demonstrates how SNOMED CT supertypes and subtypes are included or excluded using the Helper Tool.


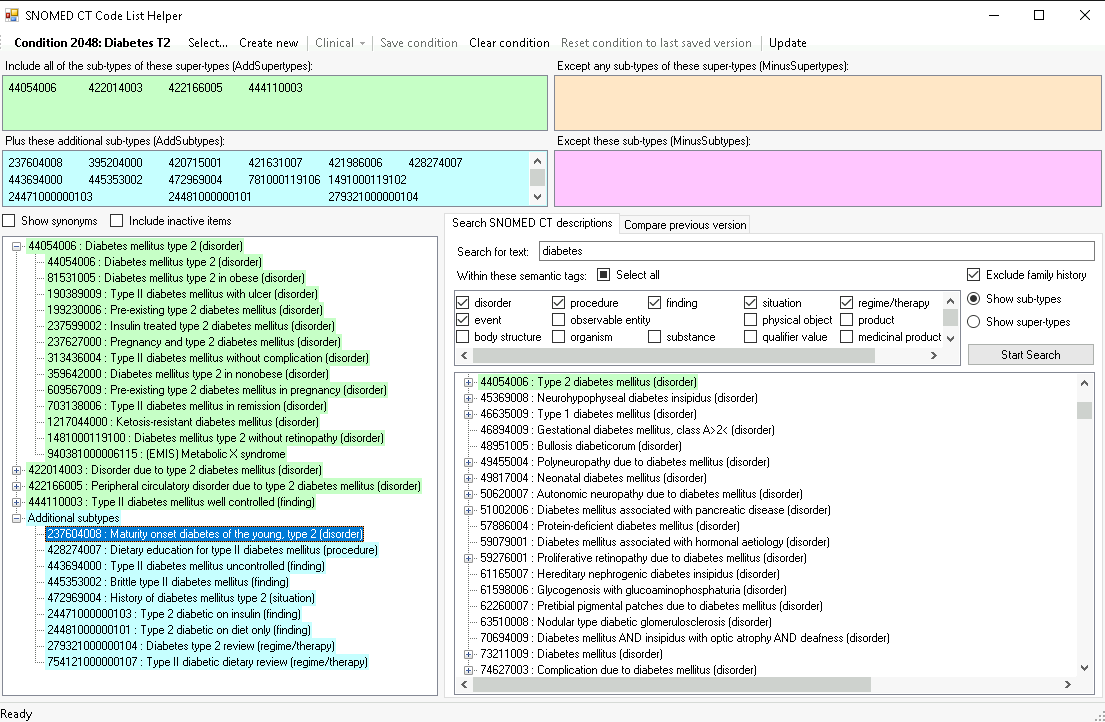

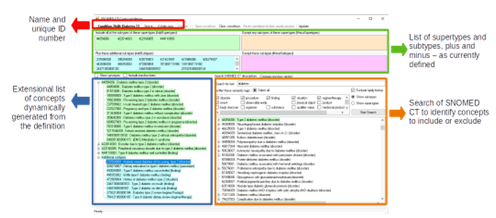

Supplement: ooae034_Supplementary_Data [file ooae034_supplementary_data.zip › Supplementary Figure S1.docx]
