# Supplementary material for: Phenotype execution and modeling architecture to support disease surveillance and real-world evidence studies: English sentinel network evaluation
Source: JAMIA Open. 2024 May 10;7(2):ooae034. doi: 10.1093/jamiaopen/ooae034 (PMC11087727; doi:10.1093/jamiaopen/ooae034)
Supplement: ooae034_Supplementary_Data [file ooae034_supplementary_data.zip › T2Diabetes.cql.docx]

/**

* Orchid Phenotype

*

* Name : Type 2 diabetes

* ID : 1

* Url : https://orchid.phc.ox.ac.uk/phenotype/137

*/

library "Type 2 diabetes" version '1.0.0'

using FHIR version '4.0.0'

include FHIRHelpers version '4.0.0' called FHIRHelpers

include PhEMAHelpers version '1.0.0' called PhEMAHelpers

valueset "Type 2 diabetes": '2048: Diabetes T2'

valueset "Diabetes Resolved": '7230: DiabetesResolved'

define "Is Resolved":

exists ([Condition:'Type 2 diabetes']) and

exists ([Condition:'Diabetes Resolved']) and

(PhEMAHelpers."Latest Date"(PhEMAHelpers."Effective Dates"([Condition:'Type 2 diabetes'])) < PhEMAHelpers"Latest Date"(PhEMAHelpers."Effective Dates"([Condition:'Diabetes Resolved'])))

define "Case" :

exists ([Condition:'Type 2 Diabetes']) and

not "Is Resolved"
